# Our evolving science: studying the influence of sex in preclinical research

**DOI:** 10.1186/s13293-016-0068-8

**Published:** 2016-02-25

**Authors:** Carolyn M. Mazure

**Affiliations:** Department of Psychiatry, Women’s Health Research at Yale, Yale University School of Medicine, 135 College Street, Suite 220, New Haven, CT 06510 USA

**Keywords:** Sex differences, Preclinical research, Laboratory research

## Abstract

The policy announcement by the National Institutes of Health that sex should be considered as a relevant variable in preclinical research has sparked considerable debate. This debate has largely centered on specific concerns regarding how the policy will be implemented. However, others have reacted to the new policy by calling into question the capacity of preclinical science to generate data that can be useful to human health. This commentary examines the basis for this contention and maintains that it is essential to expand our scientific efforts to include the influence of sex on the biology and behavior that is studied in preclinical investigations.

## Background

Questions have arisen regarding the new National Institutes of Health policy requiring grant applicants to consider sex as a variable in preclinical research. The public debate has focused largely on the specifics of how the policy will be implemented [1] and whether the requirements go too far or not far enough [2, 3]. In contrast, a recent opinion in the Proceedings of the National Academy of Sciences (PNAS) [4] uses the new policy to launch an argument highly unsupportive of preclinical research and calls into question the value of studying sex differences in preclinical science as well as the products of basic science.

In their PNAS opinion, Richardson and co-authors [4] make the important point that human health is determined by complex interactions of biology and behavior in a gendered world. They use this valid premise to protest the new National Institutes of Health (NIH) policy that female animals and cells be considered in preclinical research because such studies cannot capture the human experience. The authors provide some examples of possible pitfalls and challenges in studying sex differences in preclinical research as reasons why it is not yet time to correct our overreliance on male models. They conclude by asserting their support of preclinical research on sex differences—just that it not be required of government-funded research because preclinical research models are not validated to study sex differences and because dollars would be better spent on human studies of the influence of sex and gender on health outcomes.

## Discussion

Despite initially offering a premise with which we can readily agree, Richardson and colleagues build their argument for not supporting government-funded sex difference preclinical research on examples and assertions that do not reflect an understanding of the practice of science.

In attempting to debunk the notion that the study of female animals could lead to increased knowledge of women’s health outcomes, they point to an “unprecedented” move by the FDA in which the recommended dosage of the sleep aid zolpidem was reduced for women because adverse effects were more commonly reported by women than men. They assert that advocates of sex differences research would claim that preclinical studies would have identified the potential for greater adverse effects in females. They disagree, claiming that such reports are likely due to the use of polypharmacy by women and greater sensitivity to reporting, rather than to sex differences that could be uncovered in preclinical research. To prove their point, they state that Greenblatt et al. [5] showed sex differences in body weight accounting for slower clearance of zolpidem in women and that no difference in adverse effects was found between males and females when controlling for weight. However, Greenblatt et al. report that there are sex effects in the pharmacodynamics of the drug (the behavioral response to drug concentrations) which could have been evaluated in preclinical studies. These effects result in sex differences in the time-course and intensity of the effects of zolpidem, and placebo-normalized differences show reduced perceptual processing and reaction time for women as assessed with quantitative measures. Although these particular effects were not tested as traditional adverse effects, they nevertheless are unintended detrimental effects and definitively show sex differences that underlie the need for reduced dosing in women.

The authors further assert that “studying sex in cell lines is also far from straightforward.” This is well known and certainly acknowledged in the new NIH grant application guidelines [6] that state “It is important that researchers using cells consider the possible role of sex in their research. However, NIH recognizes current challenges to the authentication of cell lines.” As Klein et al. [7] point out, because immortalized cell lines may have become chromosomally unstable, it may not be feasible to discern the sex of the original cell. Yet, this is not the case for primary cells, and Klein et al. provide examples of sex differences reliably derived from studying primary cell lines, including demonstration of faster regeneration of new skeletal muscle cells using stem cells from females vs. males and athero-protective capabilities of bone marrow mononuclear cells derived only from female cells. Moreover, the fact that there are challenges in ensuring the sex of cell lines [8] does not reduce the importance of studying the effect of sex. The sex of cells affects their biology [9], and as Taylor et al. [10] point out, “The complement of sex chromosomes in cells studied in culture has the potential to affect expression of proteins and ‘mechanistic’ signaling pathways.”

The authors then indicate that animal studies may not effectively model the “environment” of the adult male or female “both for hormonal-milieu and gender-contextual reasons.” They illustrate this concern by citing “a non-sex related source of variation” in their report of the findings by Prendergast et al. [11] indicating that group vs. individual caging is a source of variation that can affect health behaviors. They do not report that Prendergast et al. also found there were sex differences in individual vs. group housing in that male mice housed in groups fight among themselves but females housed in groups do not. They also seem surprised that the health behaviors of male and female mice, like humans, are affected by the social environment. However, this has long been known and continues to be a target of investigation in studying hormonal effects in males [12] and comparing health outcomes in males vs. females in a diversity of disciplines [13].

The objections advanced by the authors that preclinical models are not validated to study sex differences and that dollars are better spent on human studies directly engage two questions, namely, what is the purpose of an animal model and do such models provide valid data.

The purpose of a model in preclinical research is to serve as a template for investigating what may underlie human health. Such models are not expected to have lockstep relationships with human outcomes, nor are they expected to be an exact replication of the differences found between “human men and women” as Richardson et al. suggest. Rather, models are expected wherever possible to approximate or include variables that are suspected to affect human health in order to come closer to a useful understanding of the processes involved in health and disease. The validity of an animal model is determined if its findings can translate to our understanding of human health. In fact, findings of sex differences in preclinical research have revealed important information for clinical practice as pointed out by the current directors of the Office for Research on Women’s Health and the National Institutes of Health [14]. This is not to say that all animal models have been adequately designed to ensure appropriate translation. But, here too, we see the importance of the new NIH policy, which requires grant application review committees to evaluate consideration of the influence of sex in the study design and the interpretation of results. This new policy will help to ensure that investigators develop valid models because the NIH continues to be the largest single funder of biomedical science [15]. And as such, its guidelines affect the direction, design, and implementation of research.

Additionally, the authors do not recognize that many questions can only be answered by preclinical studies due to the nature of these investigations. And because the results of these studies form the biological basis for human health studies and derivative treatments [16], it is essential to explore the effect of sex. Contrary to the claims of these authors that “the new policy’s focus (is) on nonhypothesis-driven documentation of sex differences in basic laboratory research,” the NIH grant application guidelines explicitly state that applicants should consider the variables “relevant to the experimental design of the study” and provide “sex, age, weight, and underlying health conditions” as pertinent examples [6].

## Conclusion

Rather than discourage preclinical science from embracing sex difference investigations because there may be challenges, thus repeating the missed opportunities we experienced before the inclusion of women and minorities in clinical research [17], let us encourage scientists to consider how best to develop research and educational and monitoring strategies to improve the sex balance in our studies [18]. And as McCullough and colleagues point out [18], let us develop a consensus to implement the new policy so that “science will move forward in a productive and effective manner.” I would think that Richardson et al. as well as all of us in the scientific community would want to know as much as possible about how being female might affect study outcomes. And neither assume that human studies alone can reveal what we need to know about biology and behavior nor assume that male models are sufficient to inform this search.

## Abbreviations

NIH: National Institutes of Health
PNAS: Proceedings of the National Academy of Sciences
